# Spontaneous Chiral Resolution of a Mn^III^ Spin‐Crossover Complex with High Temperature 80 K Hysteresis

**DOI:** 10.1002/chem.202300275

**Published:** 2023-05-09

**Authors:** Conor T. Kelly, Ross Jordan, Solveig Felton, Helge Müller‐Bunz, Grace G. Morgan

**Affiliations:** ^1^ School of Chemistry University College Dublin Belfield, Dublin 4 Ireland; ^2^ Centre for Quantum Materials and Technologies School of Mathematics and Physics Queen's University Belfast Belfast BT7 1NN UK

**Keywords:** chiral resolution, circular dichroism, electronic structure, spin crossover, X-ray diffraction

## Abstract

Non‐centrosymmetric spin‐switchable systems are of interest for their prospective applications as magnetically active non‐linear optical materials and in multiferroic devices. Chiral resolution of simple spin‐crossover chelate complexes into the Δ and Λ forms offers a facile route to homochiral magnetic switches, which could be easily enantiomerically enriched. Here, we report the spontaneous resolution of a new hysteretic spin‐crossover complex, [Mn^III^(sal_2_323)]SCN ⋅ EtOH (**1**), into Δ and Λ forms, without the use of chiral reagents, where sal_2_323 is a Schiff base resulting from condensation of 1,2‐bis(3‐aminopropylamino)ethane with 2‐hydroxybenzaldehyde. The enantiopurity of the Δ and Λ isomers was confirmed by single crystal X‐ray diffraction and circular dichroism. Quantum chemistry calculations were used to investigate the electronic structure. The opening of a wide 80 K thermal hysteresis window at high temperature highlights the potential for good magneto‐optical function at ambient temperature for materials of this type.

## Graphical abstract

Spontaneous chiral resolution of the Δ and Λ isomers of a racemic mixture of a Mn^III^ spin‐crossover complex is achieved by conglomerate crystallization. The complex exhibits gradual spin crossover with a wide thermal hysteresis, ∼80 K at its maximum, with *T*_1/2_↑=250 K and *T*_1/2_↓=200 K. Single‐crystal X‐ray diffraction and circular dichroism was used to confirm the enantiopurity of the Δ and Λ isomers.

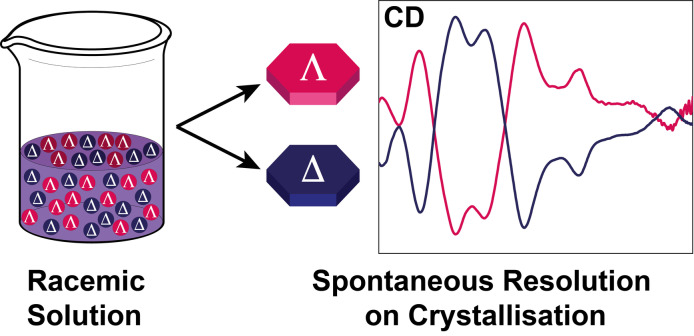

## Introduction

Spin‐crossover (SCO) complexes are well studied materials which can exhibit bistability between different spin states,^[1]^ most often between the low spin (*S*=0) and high spin (*S=*2) state of Fe^II^,^[2]^ but now also well reported in Fe^III^,^[3]^ Mn^III^,^[4]^ Cr^II^,^[4a]^ and Co^II^.^[4a]^ The large changes in physical properties that occur with changing spin state can be exploited in the development of chemical sensors,^[5]^ molecular actuators,^[6]^ responsive magnetic resonance imaging (MRI) contrast agents^[7]^ and nano thermometry devices.^[8]^ Chirality – which plays an important role in many fields including medicine,^[9]^ biology,^[10]^ and catalysis^[11]^ ‐ is also important in magnetism^[12]^ particularly in the development of new non‐linear optical (NLO) materials^[13]^ and in spintronic devices with spin polarized electrons.^[14]^ Although the majority of SCO materials are centrosymmetric, there is growing interest in those which crystallize in non‐centrosymmetric enantiomorphic space groups.^[15]^ In some cases this occurs by spontaneous chiral resolution,[^4e^, ^4f^, ^4g^, ^16^] but in the main, enantiopure SCO samples have been targeted by the use of chiral ligands^[17]^ or chiral anions.^[18]^

**Figure 1:**
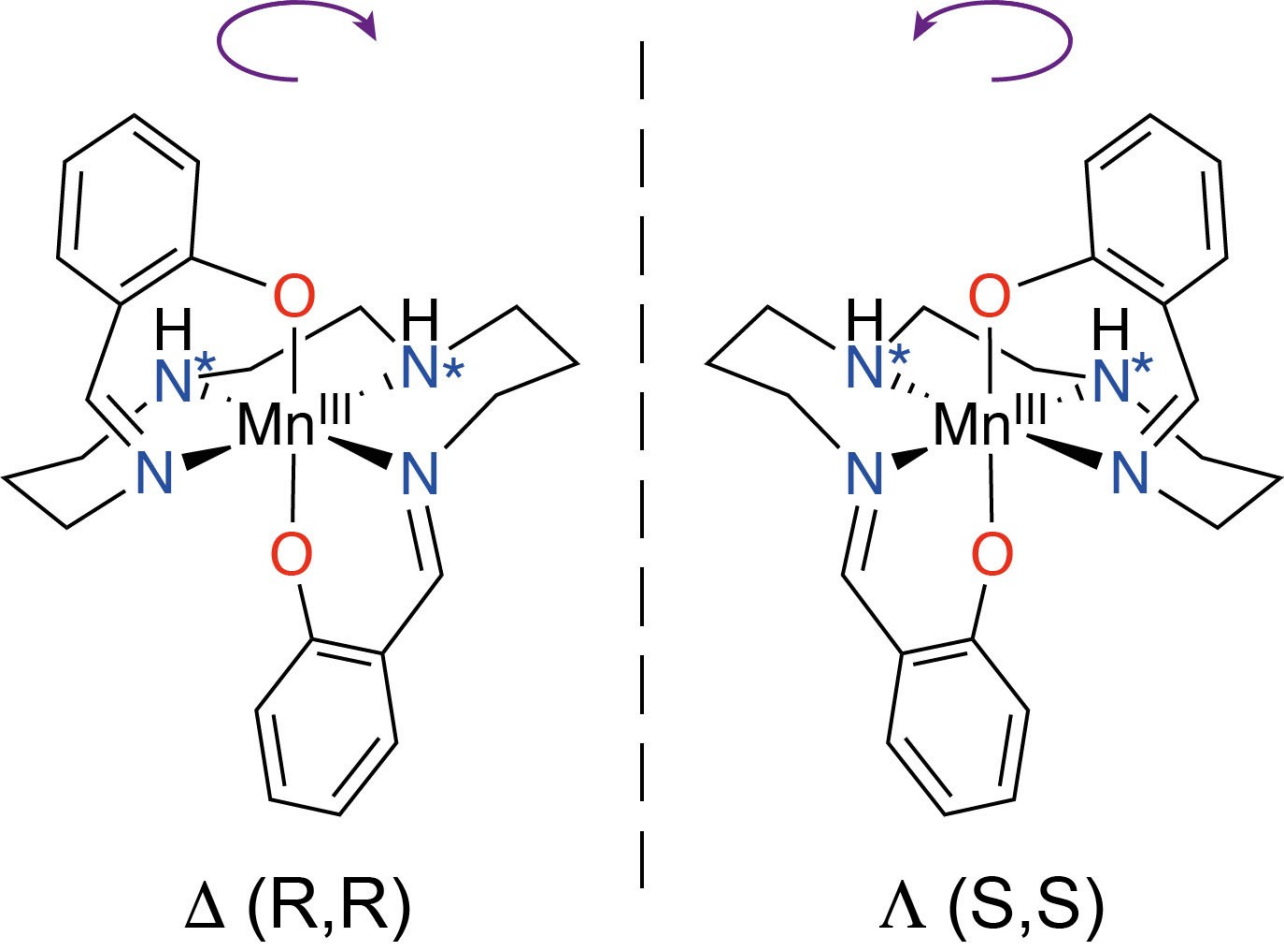
General structure of [Mn^III^(sal_2_323)]^+^ complexes showing the Δ and Λ enantiomers which are so named due to the twisting of the ligand around the Mn^III^ center, in either a clockwise or anticlockwise direction. The amine nitrogens of the backbone (*) are chiral centers where the configuration is *R,R* (Δ) or *S,S* (Λ).

Use of an achiral chelating ligand confers chirality at the metal center by twisting around the ion in a clockwise (Δ) or anti‐clockwise (Λ) fashion and this chelate type is well known in SCO systems including the *R*‐sal_2_323 ligand series which promotes thermal spin state switching in Mn^III^,^[4a]^ Figure 1.

While chiral resolution of [Mn^III^(*R*‐sal_2_323)]^+^ SCO complexes has been targeted by use of chiral anions^[18b]^ we have recently noted the preference for homochiral crystallization when the 4‐OMe‐sal_2_323 ligand is used without any other chiral agent.^[16a]^ This suggests a ligand directed effect in the crystal packing, and analysis of the wider library of [Mn^III^(*R*‐sal_2_323)]^+^ compounds indicates that the unsubstituted sal_2_323 ligand may also promote homochiral crystallization via a ligand effect. This is evidenced by the spontaneous chiral resolution of the ClO_4_^−^,^[19]^ ReO_4_^−^,^[4g]^ PF_6_^−^,^[4e]^ and AsF_6_^−^,^[4f]^ salts of the [Mn^III^(sal_2_323)]^+^ complex, i. e., when crystallized with anions which are tetrahedral or octahedral. We now report the magnetic and structural properties of new SCO complex, [Mn^III^(sal_2_323)]SCN ⋅ EtOH (**1**), with a simple linear thiocyanate anion, which also shows spontaneous chiral resolution and a wide thermal hysteresis at room temperature. We also discuss here the factors which may influence the preference for chiral crystallization of some [Mn^III^(*R*‐sal_2_323)]^+^ SCO complexes.

## Results and Discussion

The [Mn^III^(sal_2_323)]SCN ⋅ EtOH complex, **1**, was synthesized by using a facile one‐pot Schiff base condensation followed by complexation around a manganese center, Scheme S1.1. The complex crystallizes in the Sohncke space group, *P*2_1_, as either the **1‐Δ** or **1‐Λ** isomer, Figure 1. Spontaneous resolution of the racemic solution of the complex occurs with the crystallization of enantiopure crystals containing either the **Δ** or **Λ** isomer. The complexes **1‐Δ** and **1‐Λ** are isostructural except for the inversion of the chirality. The absolute structures of **1‐Δ** and **1‐Λ** were assigned unambiguously using SCXRD, with Flack,^[20]^ Hooft^[21]^ parameters and Parsons’ quotient^[22]^ close to zero in all cases, Table 1.

**Table 1:** Bond lengths and distortion parameters of **1‐Δ** and **1‐Λ** at variable temperatures.

Complex	1‐Δ		1‐Λ	
*T* (K)	100(2)	293(2)	100(2)	310(4)
Bond Lengths (Å)				
Mn−O	1.8805(18)	1.874(2)	1.8800(15)	1.885(2)
	1.8840(18)	1.883(2)	1.8852(15)	1.877(2)
Mn−N_imine_	1.991(2)	2.052(3)	1.9924(18)	2.058(3)
	1.995(2)	2.036(3)	1.9943(17)	2.047(3)
Mn−N_amine_	2.044(2)	2.106(3)	2.0432(18)	2.137(3)
	2.052(2)	2.123(3)	2.0530(18)	2.119(3)
				
Flack^[20]^	−0.002(2)	−0.004(3)	−0.008(2)	−0.004(2)
Hooft^[21]^	−0.0040(14)	0.000(2)	−0.0060(12)	0.0049(13)

Distortion parameters (°)				
Σ	27.85	40.78	27.91	43.40
Θ	86.40	130.40	86.45	138.77

### Single‐crystal X‐ray diffraction

**Figure 2:**
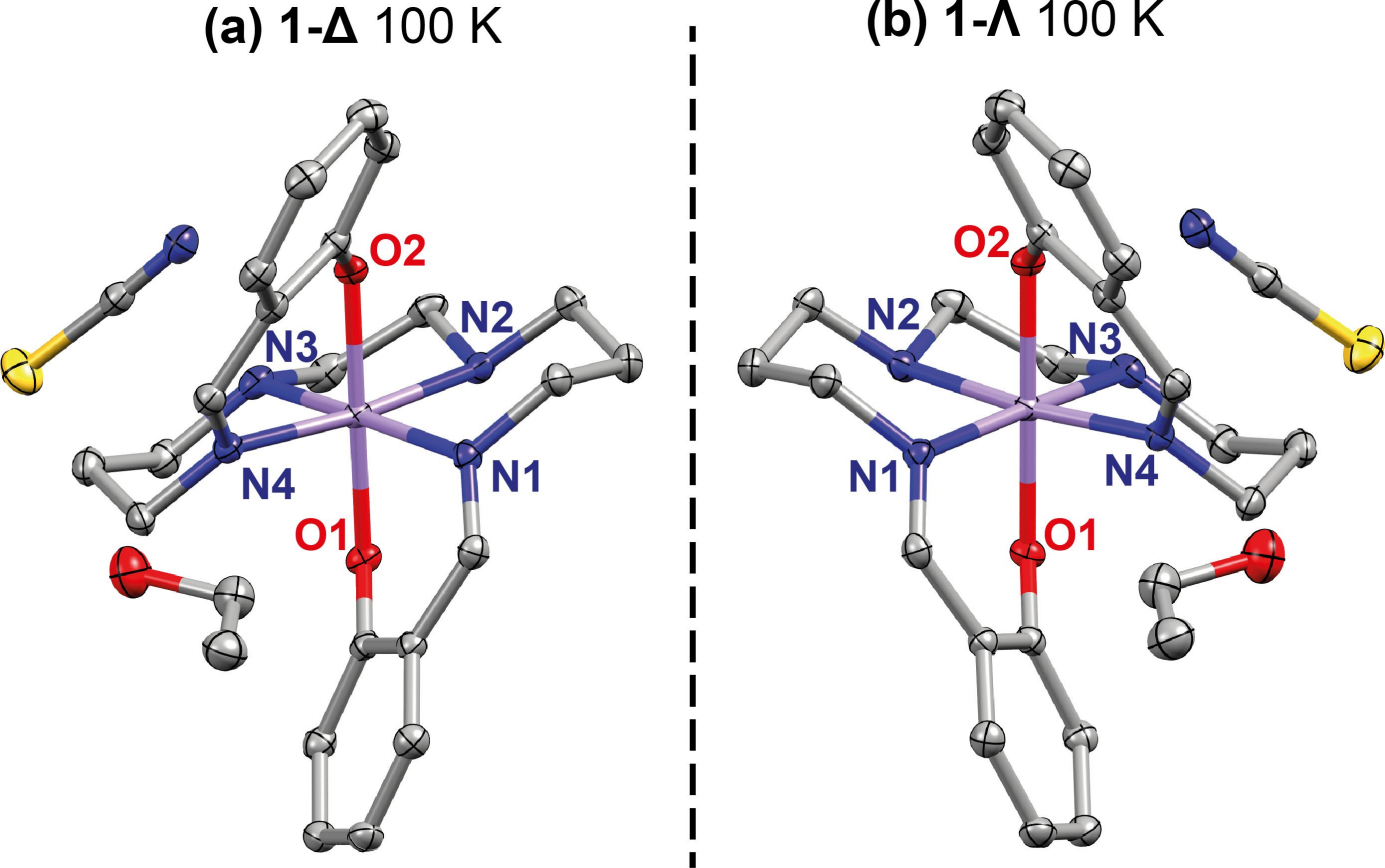
Asymmetric unit of a) **1‐Δ** and b) **1‐Λ** at 100 K. Ellipsoids are drawn at 50 % atomic probability. Hydrogen atoms have been omitted for clarity.

The structures of both **1‐Δ** and **1‐Λ** were determined at 100 K and at higher temperature in the high‐spin (HS) regime. In both cases the asymmetric unit contains one cation, one anion and one ethanol molecule, Figure 2. The bond lengths around the Mn^III^ center are indicative of the spin state of the complex, with shorter bond lengths at 100 K representative of the LS (*S*=1) state and longer bond lengths at room temperature representative of the HS (*S*=2) state, Table 1. Octahedral distortion parameters Σ and Θ,^[23]^ are also used to quantify the degree of distortion around the Mn^III^ center with differing spin states, Table 1. The values for Σ and Θ are larger for the HS state due to an increased distortion.

**Figure 3:**
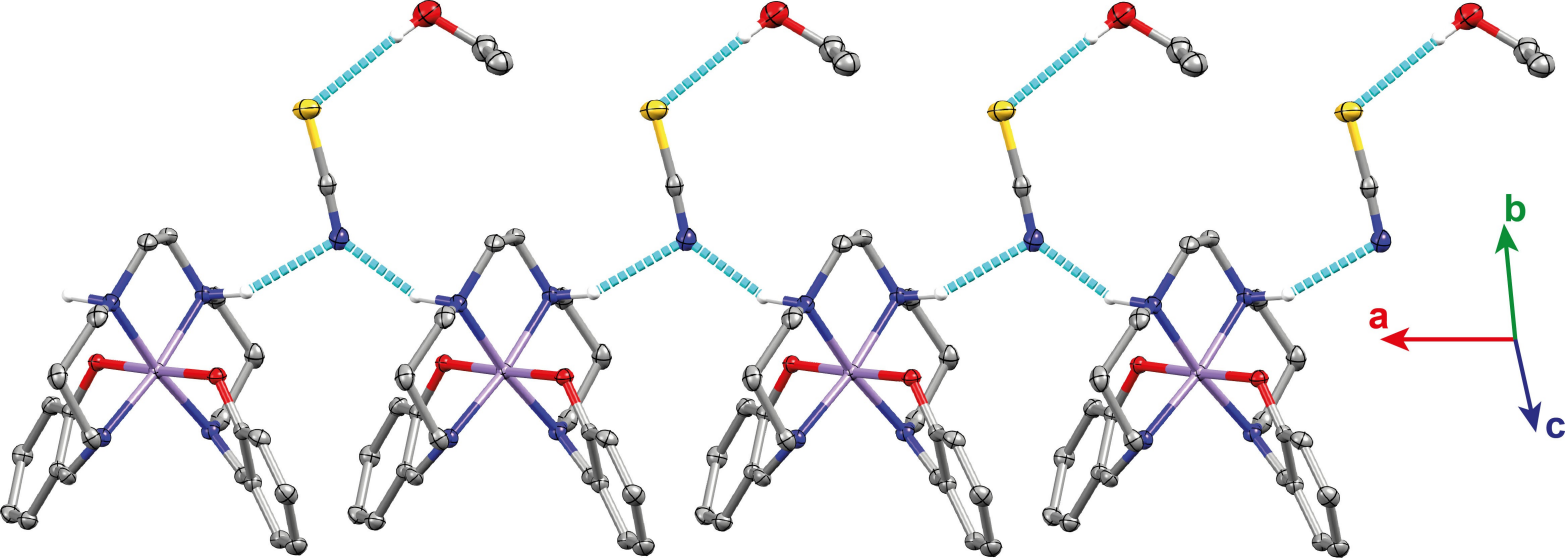
Intermolecular hydrogen bonding chain formation for **1‐Λ** at 100 K between the amines of the backbone of the complex and the thiocyanate anion. Additional anion‐solvent hydrogen bonds are observed.

The formation of intermolecular hydrogen bonding chains is observed, connecting the amine nitrogen atoms on the backbone of the ligand with the thiocyanate anion, Figure 3. Further details for the hydrogen bonds can be found in Table S2.3. A lengthening of the hydrogen bonds is observed upon increasing the temperature. Hirshfeld surface analysis^[24]^ was also performed on each structure of **1‐Δ** and **1‐Λ** using the CrystalExplorer 21 software.^[25]^ This analysis reveals additional C−H⋅⋅⋅C and C−H⋅⋅⋅S interactions that are shorter than the van der Waals radii of the respective atoms, Figure S2.5 and S2.6. The C5‐H5⋅⋅⋅S interaction occurs between a hydrogen of the phenolate ring and the thiocyanate anion. For **1‐Δ** the C5‐H5⋅⋅⋅S distance increases from 3.774(3) Å to 3.803(4) Å upon increasing the temperature from 100 K to 293 K.

**Figure 4:**
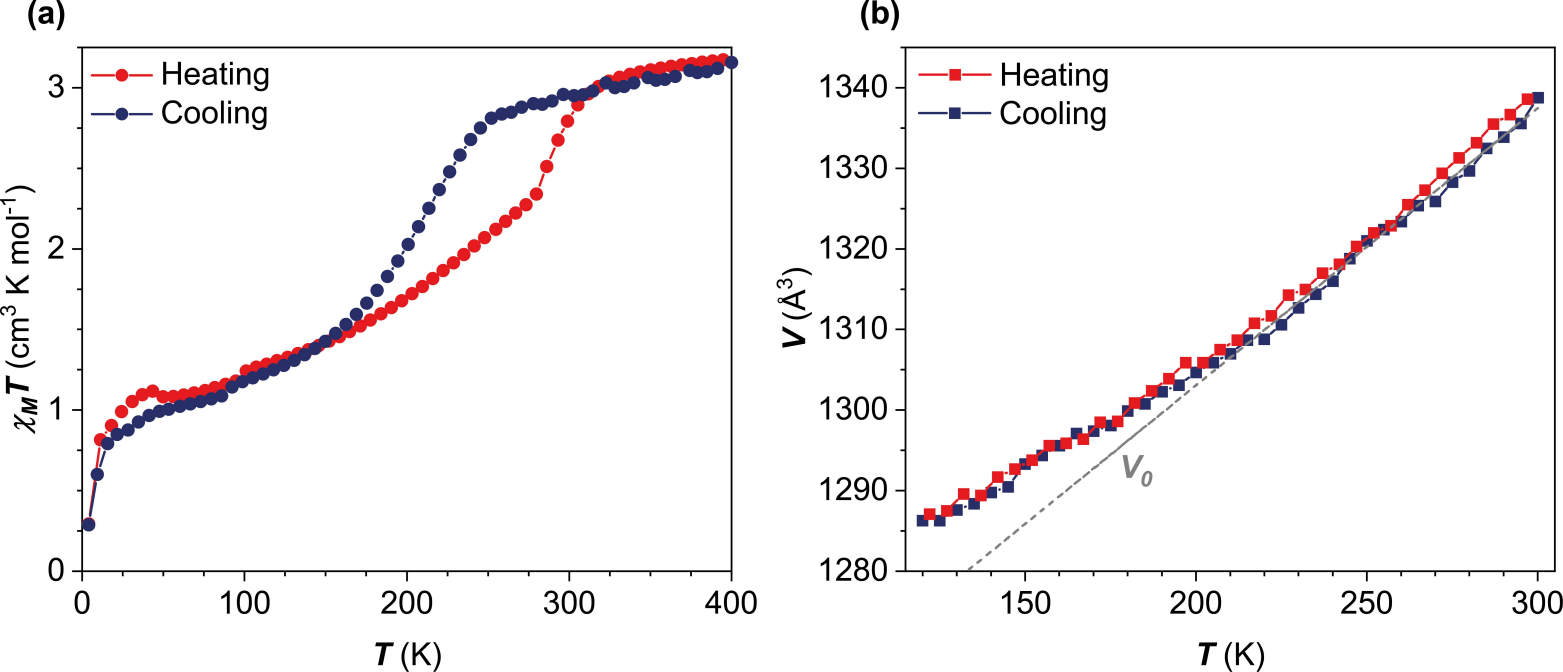
a) *χ_M_T* data for **1** recorded between 4 K and 400 K at a scan rate of 1 K min^−1^. b) Change in unit cell volume, *V*, between 300 K and 120 K recorded in heating and cooling modes. Linear fit of the higher temperature data points gives the extrapolated dashed grey line, *V*_0_. The data was integrated with respect to the high temperature *P*2_1_ cell. Errors are smaller than the size of the data points. Further unit cell parameters can be found in Figure S2.3.

Variable‐temperature determination of the unit cell parameters was used to track the SCO in **1** between 120 K and 300 K in both heating and cooling modes. A discontinuity in unit cell parameters is observed at *T_c_=*213 K for both heating and cooling modes and is shown for the unit cell volume, *V*, in Figure 4(b). The equivalent plots for the other unit cell parameters are shown in Figure S2.3. We do not observe a hysteresis in the changing of the unit cell parameters, and the changes occur at 213 K in both heating and cooling modes.

With respect to the spontaneous chiral resolution of **1‐Δ** and **1‐Λ**, we note that of the 129 [Mn^III^(*R*‐sal_2_323)]^+^ complexes reported with structures deposited in the Cambridge Structural Database (CSD) only 18 crystallize in Sohncke space groups (14.0 %), Table S2.5.[^4c^, ^4e^, ^4f^, ^4g^, ^18b^, ^19^, ^26^] Three of these structures contain both the Δ and Λ enantiomers as independent sites in the asymmetric unit.[^4c^, ^26c^, ^26d^] Enantiopure forms of both the Δ and Λ isomers of the [Mn^III^(5‐OCF_3_‐sal_2_323)]^+^ complex were recovered by using a chiral anion, (*R,R*) or (*S,S*)‐bis(1,1’‐binaphthyl‐2,2’‐diolate)boron.^[18b]^ That leaves 13 complexes of the [Mn^III^(*R*‐sal_2_323)]^+^ form whereby resolution of Δ and Λ enantiomers by conglomerate crystallization is possible, and within this we observe a preference with ligands derived from salicylaldehyde and 4‐methoxysalicylaldehyde.

### Magnetic measurements

The magnetic susceptibility of **1** was recorded between 4 K and 400 K, Figure 4(a) on a polycrystalline sample. The measurement reveals a gradual *S*=2↔*S=*1 transition, with a reduction of the *χ_M_T* value of 3.03 cm^3^ K mol^−1^ at 300 to 1.01 cm^3^ K mol^−1^ at 50 K. The sharp downturn in the *χ_M_T* value at low temperature can be attributed to zero field splitting. The transition is hysteretic with *T*_1/2_↑=250 K and *T*_1/2_↓=200 K, i. e. a 50 K hysteresis using the T_1/2_ values. The asymmetry of the thermal evolution of the *χ_M_T* versus *T* data, however, results in a wider hysteresis loop of 80 K at higher temperatures. This value was obtained from the first derivative *χ_M_T /dT* values following the method of Garcia et al. for asymmetric SCO profiles.^29d^ The derivative values, Figure S3.1, show maxima at *T*_hy_↓=213 K and *T*_hy_↑=293 K, where *T*_hy_ is the temperature at the extremes of the hysteresis window, i. e. a hysteresis window of 80 K at the widest point in the data for complex **1**.

Thermal hysteresis is often observed in abrupt SCO systems,^[27]^ when it may be coupled to a symmetry‐breaking structural phase transition,^[28]^ but hysteretic gradual SCO is also known.^[29]^ No structural symmetry‐breaking was detected in **1** and while the hysteresis is not clearly replicated in the change in lattice parameters, Figure S2.3, there is a slight change in the slope around 213 K. This temperature matches the *T_hy_* ↓ value obtained from the magnetic susceptibility measurement. Whilst thermal hysteresis in Mn^III^ SCO is known,[^4b^, ^4c^, ^4d^, ^4e^, ^4f^, ^4g^, ^4h^, ^4i^] it is rare, with the most striking example being that of the [Mn^III^(5‐Cl‐sal_2_323)]TCNQ_1.5_ ⋅ 2CH_3_CN complex which exhibits a 50 K hysteresis window centered at 100 K with an abrupt transition.^[4b]^ [Mn^III^(sal_2_323)]SCN ⋅ EtOH (**1**) is, however, the first example with opening of a wide hysteresis window close to ambient temperature.

### Optical spectroscopy

**Figure 5:**
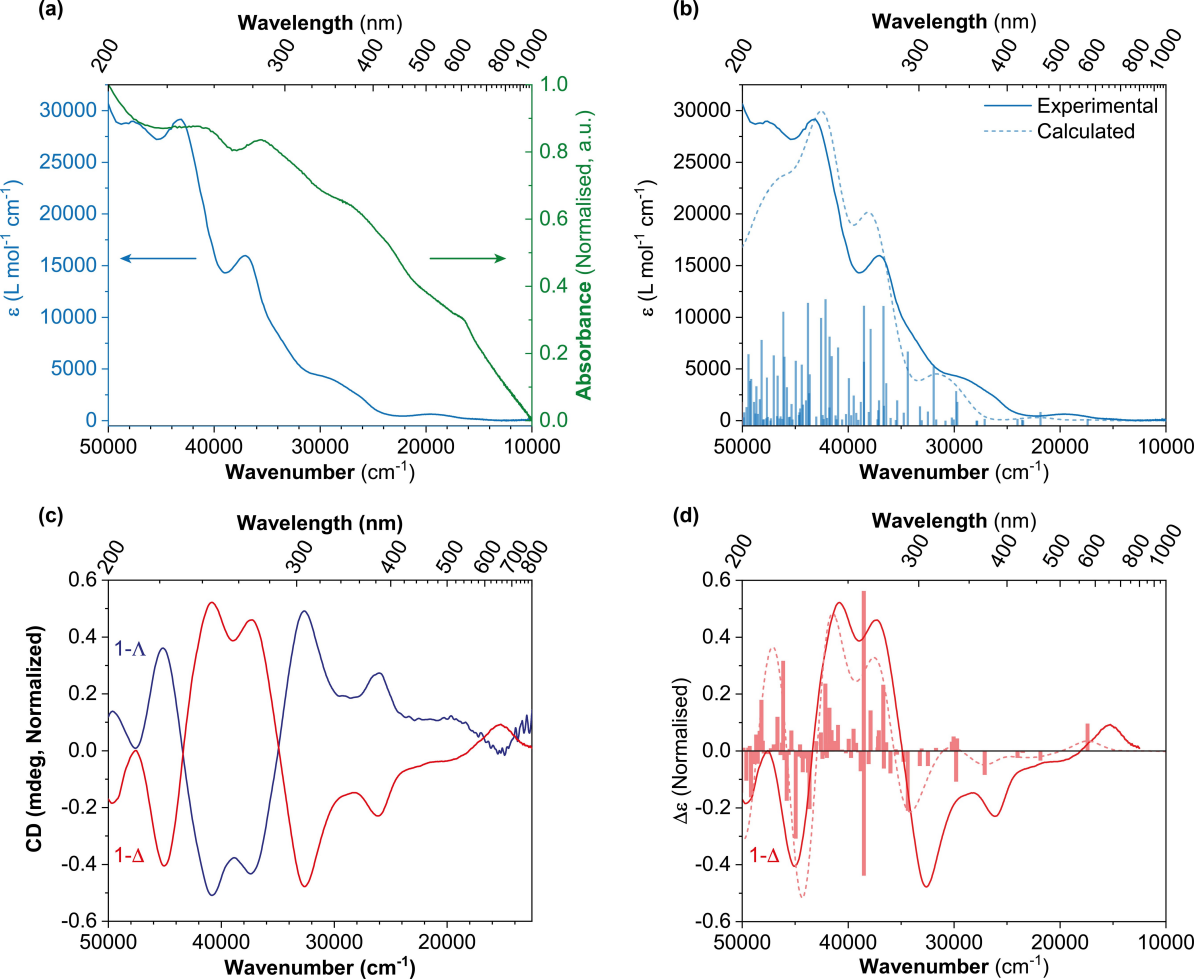
a) Absorbance spectra of **1** recorded in acetonitrile (blue) and as a solid‐state mull in silicone oil (green). b) Absorbance spectrum of **1** in acetonitrile with calculated transitions using TD‐DFT (CAM‐B3LYP/def2‐TZVPP). c) CD spectra of both **1‐Δ** (red) and **1‐Λ** (navy) isomers recorded in a solution of acetonitrile, with a band width of 2 nm and scan rate of 200 nm min^−1^. d) CD spectrum of **1‐Δ** with the calculated transitions using TD‐DFT (CAM‐B3LYP/def2‐TZVPP).

The UV‐visible absorption spectrum of **1** was recorded both in solution and in the solid state, Figure 5(a). Prominent bands at 212 nm (*ϵ_max_* =28,960 L mol^−1^ cm^−1^), 231 nm (*ϵ_max_* =29,100 L mol^−1^ cm^−1^), and 270 nm (*ϵ_max_* =15,900 L mol^−1^ cm^−1^) are observed in acetonitrile solution. A shoulder at 340 nm (*ϵ_max_* =4,230 L mol^−1^ cm^−1^) and a weak band at 508 nm (*ϵ_max_* =630 L mol^−1^ cm^−1^) are also observed. We attribute the band at 212 nm to a π to π* transition in the aromatic moiety of the ligand, the bands at 231, 270 and 340 nm to LMCT transitions, and the weak band at 508 nm to a *d‐d* transition, which we assign using TDDFT, see below. In the solid‐state mull of **1** we observe broad bands at *λ_max_* =241 nm, 281 nm, 370 nm, and 600 nm. The solid‐state spectrum is broader and more shifted, presumably due to scattering and absorption flattening because of inhomogeneous particle size distribution in the silicone mull.^[30]^

Circular dichroism (CD) was used to study the chirality of the different isomers, **1‐Δ** and **1‐Λ**, by measuring the difference in the absorbance of left‐ and right‐handed circularly polarized light as a function of wavelength, Figure 5(c). The experiments were performed by taking a single crystal of **1** and splitting it in two pieces, dissolving one piece in acetonitrile and recording the CD spectrum and taking the other piece to measure SCXRD to appropriately assign the absolute structure to the CD spectrum. For **1‐Δ** a positive Cotton effect is observed at 245 nm, 268 nm and 654 nm, and a negative Cotton effect at 220 nm, 307 nm and 383 nm. For **1‐Λ** the exact opposite Cotton effect is observed at the same wavelengths. We also confirmed the assignment with TDDFT, see below. Racemization of the complex in solution does not occur over the timescale of the experiment.

### Quantum chemistry calculations

Time‐dependent density functional theory (TDDFT) was used to evaluate the excited state properties of **1** using the ORCA 5.0.1 software package.^[31]^ The optimized structure of the HS (*S=*2) cation, [Mn^III^(sal_2_323)]^+^, was used to calculate the absorbance and CD spectra, Figures 5(b) and (d). The predicted spectra were determined using TDDFT and the range‐separated hybrid CAM‐B3LYP functional^[32]^ and the fully polarized def2‐TZVPP basis set.^[33]^ Solvation effects were accounted for with the CPCM solvation model^[34]^ (acetonitrile, ϵ=37.5) as implemented in ORCA. Simulated spectra were shifted to get an optimal fit with the experimental spectra, performed using the SpecDis program.^[35]^ The similarity factor was determined to be 0.99 for the UV‐vis absorbance spectrum and 0.80 for the CD spectrum with a 17 nm shift of the spectra. The important transitions are summarized in Table S4.3. Difference density surfaces for a selected number of transitions are shown in Figure S4.1.

Quantum chemistry can also be used to determine the difference in Gibbs free energy between the HS and LS states, *ΔG_SCO_*. We use the procedure developed by Kepp,^[36]^ whereby the electronic energy difference, *ΔE*, is calculated with the B3LYP* (15 % HF exchange) functional and the def2‐TZVPP basis set. Thermodynamic corrections to the Gibbs free energy, *ΔG_therm_*, were determined by thermochemistry calculation of the optimized geometry at the BP86/def2‐TZVP level. A *ΔG_SCO_* value of −11.81 kJ mol^−1^ was obtained for [Mn^III^(sal_2_323)]^+^, showing that the HS state is more stable, as expected for Mn^III^, but the *ΔG_SCO_* value is sufficiently small to permit thermal SCO between both states. However, these calculations negate the effect of the anion, intermolecular interactions and packing in the crystalline lattice, which are well known to drastically affect the presence and nature of SCO.

## Conclusions

We report here a new Mn^III^ SCO complex with high temperature hysteresis over an 80 K range at its widest point. Complex **1**, [Mn^III^(sal_2_323)]SCN ⋅ EtOH, was recovered by crystallization of the Δ and Λ isomers from a racemic solution, i. e., spontaneous resolution by conglomerate crystallization. This fits an emerging pattern of spontaneous chiral resolution in specific Mn^III^ chelates of the [Mn^III^(*R*‐sal_2_323)]^+^ family which suggests a ligand directed effect in some cases. Although use of chiral precursors generally guarantees enantiopure complexes (as long as racemization cannot occur), they can be difficult to synthesize, are more limited due to the smaller pool of chiral reagents available and are often substantially more expensive than their achiral analogues. As such, spontaneous resolution of racemic mixtures could offer a more viable route towards chiral SCO materials through conglomerate crystallization, whereby each individual crystal contains only one enantiomer. Walsh et al. recently showed that conglomerate crystallization could potentially open up a much wider chiral pool in organic synthesis.^[37]^ Conglomerate crystallization can also be biased to give rise to enantioenrichment, using preferential crystallization^[38]^ or Viedma ripening,^[39]^ the latter method being particularly well suited to coordination compounds. Spontaneous chiral resolution may therefore offer a more facile route towards chiral SCO materials than using chiral anions or chiral ligands. Such multifunctional complexes that combine chirality and SCO are potentially of great use in the development of switchable non‐linear optical materials and spintronic devices.

## Experimental Section

**Synthesis of [Mn^III^(sal_2_323)]SCN ⋅ EtOH (1)**: Salicylaldehyde (61.1 mg, 0.5 mmol) and 1,2‐bis(3‐aminopropylamino)ethane (43.6 mg, 0.25 mmol) were mixed together in a solution of acetonitrile/ethanol (1 : 1, 10 mL), a bright yellow color was immediately observed. To this manganese(II) nitrate tetrahydrate (62.8 mg, 0.25 mmol) and potassium thiocyanate (29.2 mg, 0.3 mmol) were added. The solution was swirled to dissolve the solids, a dark purple color was observed. Dark purple blocks crystallized after days of slow evaporation of the solvent. Elemental Analysis, calculated for C_25_H_34_N_5_O_3_SMn, Theory % (Found %): C 55.65 (55.56); H 6.35 (6.25); N 12.98 (12.86). UV‐Vis (ACN) *λ_max_* nm (*ϵ_max_* L mol^−1^ cm^−1^): 212 (28960), 231 (29100), 270 (15900), 340 (4230), 508 (630). Infrared (ATR‐IR) *ν* (cm^−1^): 3431.7 (w), 3178.8(w), 2950.5(w), 2913.8(w), 2862.8(w), 2055.3(s), 1619.0(s), 1596.6(s), 1543.5(m), 1470.5(w), 1441.6(s), 1386.5(w), 1337.6(w), 1282.5(s), 1239.7(w), 1229.5(w), 1205.1(w), 1150.0(m), 1125.5(m), 1074.6(m), 1046.0(m), 1009.3(m), 984.8(m), 931.8(w), 907.4(m), 860.5(s), 813.6(w), 791.1(m), 770.7(s) 742.2(m), 644.3(w), 623.9(s), 564.8(w), 550.5(w), 491.4(w), 456.7(w), 438.4(w).

**Single‐crystal X‐ray Diffraction (SCXRD)**: SCXRD data was collected on suitable crystals of **1** using a Rigaku Oxford Diffraction SuperNova diffractometer fitted with an Atlas CCD detector. The experiments were performed using Cu−Kα radiation, with datasets collected at 100 K and at a temperature close to room temperature, Table 1. The temperature was controlled using an Oxford Cryosystems instrument. The temperature was not controlled for the room temperature measurements. *CrysAlis* ^*PRO*^ software was used for the data collection, integration, reduction and finalization.^[40]^ Complete datasets were collected assuming the Friedel pairs were inequivalent, and the absolute structure was unambiguously determined with Flack,^[20]^ Hooft,^[21]^ and Parsons’ quotient^[22]^ parameters close to zero. The structures were solved using direct methods with ShelXS^[41]^ or intrinsic phasing with ShelXT^[42]^ and refined by full matrix least‐squares on F^2^ using ShelXL.^[43]^ Hydrogen atoms were geometrically constrained and refined riding on the parent atom. All‐non hydrogen atoms were refined anisotropically. The Mercury program was used for the visualization of the structures and for the analysis of intermolecular interactions.^[44]^ Further crystallographic details can be found in Table S2.2. Hirshfeld surface analysis^[24]^ was performed using CrystalExplorer 21 software.^[25]^ Octahedral distortion parameters were determined using the OctaDist program.^[23c]^

Deposition Numbers 2217780, 2217781, 2217782, 2217783 contain the supplementary crystallographic data for this paper. These data are provided free of charge by the joint Cambridge Crystallographic Data Centre and Fachinformationszentrum Karlsruhe Access Structures service.

**Optical Spectroscopy**: Absorption spectra were recorded using an Agilent Cary 60 spectrometer. Measurements in solution were performed in acetonitrile and solid‐state measurements with the sample ground into a mull in silicone oil. CD spectra were recorded using a JASCO J‐810 spectrometer. Measurements in solution were performed by dissolving crystal(s) in acetonitrile at a concentration of 0.05 mg/mL. CD spectra were recorded with a scan rate of 200 nm/min, a band width of 2 nm and standard sensitivity (100 mdeg).

**Physical Measurements**: Infrared spectra were obtained using a Bruker Alpha Platinum ATR‐FTIR spectrometer fitted with a diamond anvil. Elemental analysis was performed using an Exeter Analytical CE 440 elemental analyzer. Magnetic susceptibility measurements were performed using a Quantum Design MPMS XL SQUID magnetometer. Polycrystalline samples were packed in a gelatin capsule. The inherent diamagnetic contribution of the sample and the gelatin capsule was corrected for using Pascal's constants.^[45]^

**Quantum Chemistry Calculations**: Theoretical quantum chemistry calculations were performed using the ORCA 5.0.1 computational package.^[31]^ The experimental SCXRD structural geometry was used as a starting point for the calculations. The structure of the cation, [Mn^III^(sal_2_323)]^+^, was optimized using the BP86 functional^[46]^ and the polarized triple ζ def2‐TZVP basis set.^[33]^ The effects of solvation were accounted for using the conductor‐like polarizable continuum (CPCM) solvation model with acetonitrile (ϵ=37.5) as the solvent.^[34]^ The atom‐pairwise dispersion correction (D3BJ) was used.^[47]^ The optimized geometries were subject to an analytical frequency calculation at the BP86‐def2‐TZVP level to ensure that the geometries represented stable energy minima and also to determine the zero‐point vibrational energies and thermodynamic correction, *ΔG_therm_*. The Cartesian coordinates for the optimized geometries can be found in Table S4.1. Single point energies were subsequently calculated on the optimized geometries using the B3LYP* functional^[48]^ (15 % HF exchange) and the fully polarized def2‐TZVPP basis set and the D3BJ dispersion correction. Spectroscopic properties were determined using TDDFT. For this the CAM‐B3LYP functional^[32]^ was used with the fully polarized def2‐TZVPP basis set^[33]^ and the CPCM solvation model. TDDFT calculations in ORCA use the Tamm‐Dancoff approximation (TDA).^[49]^ Results of the TDDFT calculations are included in Table S4.3.

## Conflict of interest

The authors declare no conflict of interest.

1

## Supporting information

As a service to our authors and readers, this journal provides supporting information supplied by the authors. Such materials are peer reviewed and may be re‐organized for online delivery, but are not copy‐edited or typeset. Technical support issues arising from supporting information (other than missing files) should be addressed to the authors.

Supporting Information

## Data Availability

The data that support the findings of this study are available in the supplementary material of this article.
