# Supplementary material for: Spontaneous Chiral Resolution of a MnIII Spin‐Crossover Complex with High Temperature 80 K Hysteresis
Source: Chemistry. 2023 May 9;29(37):e202300275. doi: 10.1002/chem.202300275 (PMC10946779; doi:10.1002/chem.202300275)
Supplement: Supplementary file 1 — Supporting Information [file CHEM-29-0-s001.pdf]

Chemistry–A European Journal

Supporting Information

Spontaneous Chiral Resolution of a Mn^{III} Spin-Crossover Complex with High Temperature 80 K Hysteresis

Conor T. Kelly, Ross Jordan, Solveig Felton, Helge Müller-Bunz, and Grace G. Morgan*

Table of Contents:

S1 Experimental Details **2**

S1.1 Synthesis of [Mn^{III}($sal_2$323)]SCN 2

S1.2 Physical Measurements 2

S2 Single Crystal X-ray Diffraction **3**

S2.1 Intermolecular Interactions 5

S2.2 Packing 5

S2.3 Variable Temperature SCXRD 6

S2.4 Hirshfeld Surface Analysis 7

S2.5 Database Analysis 10

S3 Magnetic Measurements **11**

S4 Quantum Chemistry Calculations **12**

S5 CRediT Author Statement **14**

S6 References **15**

S1 Experimental Details

S1.1 Synthesis of [Mn^{III}(sal$_2$323)]SCN

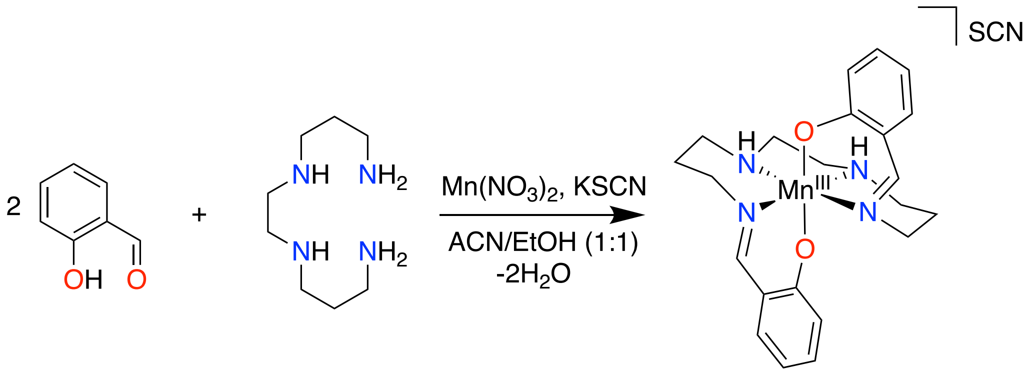

Scheme S1.1 Synthetic scheme for **1**.

S1.2 Physical Measurements

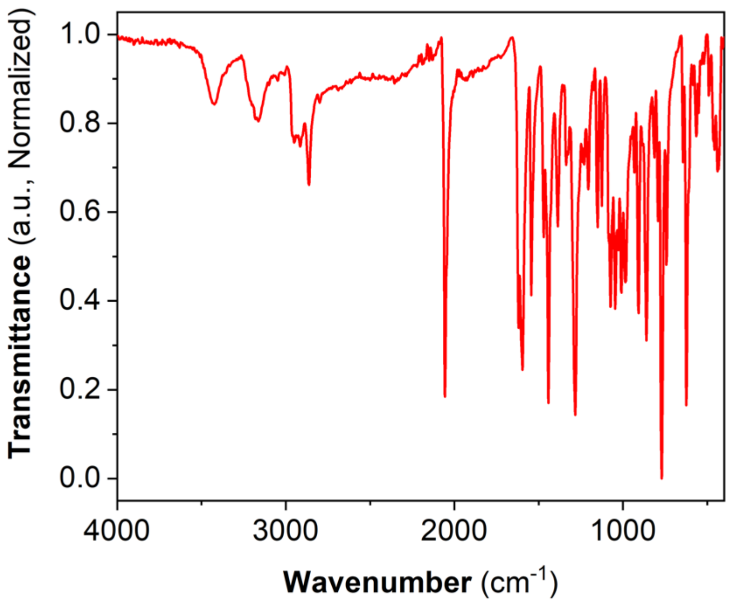

Figure S1.1 Infrared spectrum of **1** recorded on a polycrystalline sample between 400 cm^{-1} and 4,000 cm^{-1}.

S2 Single Crystal X-ray Diffraction

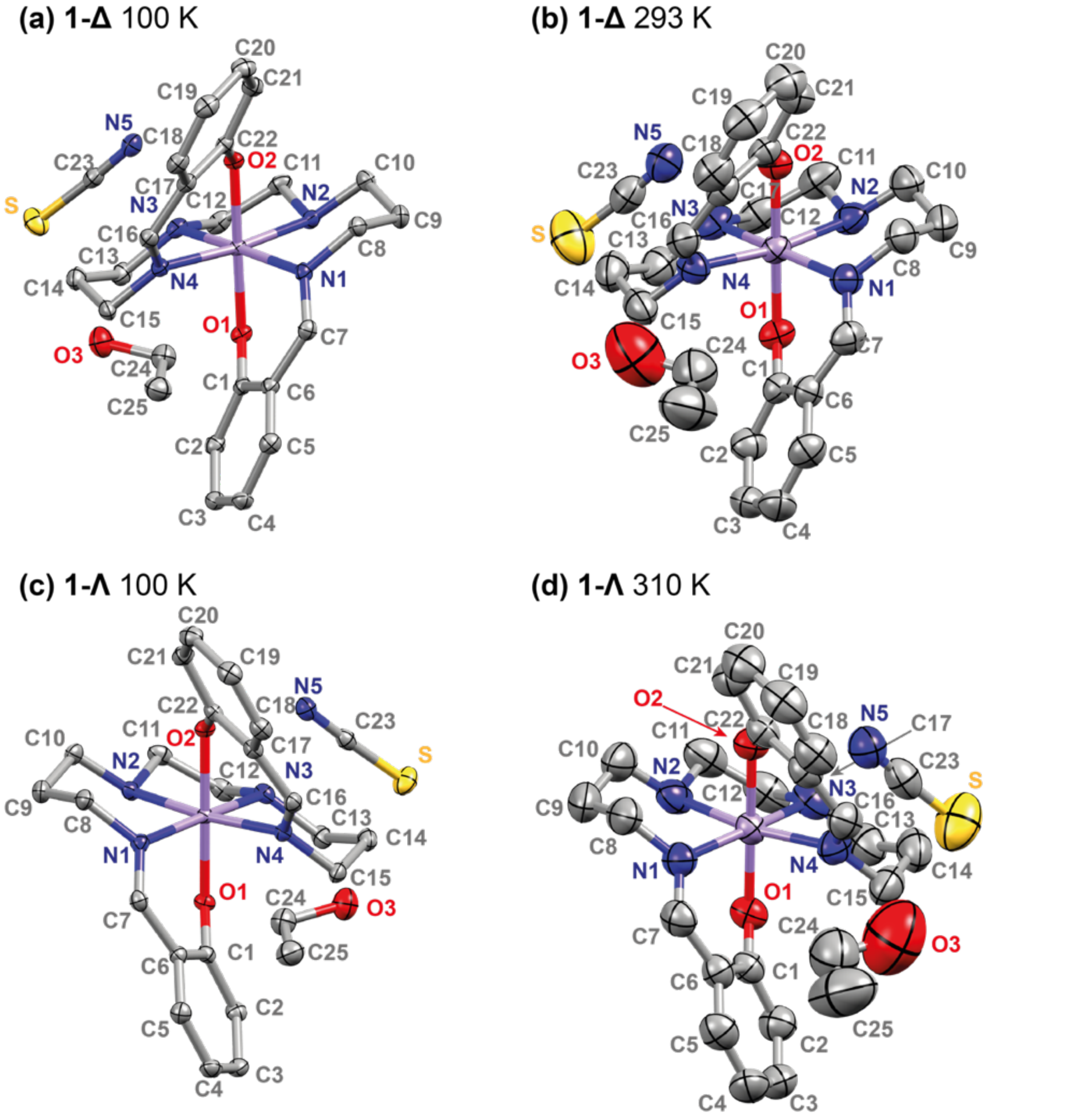

Figure S2.1 Asymmetric unit of **1-Δ** at 100 K **(a)** and 293 K **(b)** and **1-Λ** at 100 K **(c)** and 310 K **(d)**. Ellipsoids are drawn at 50% probability. Hydrogen atoms have been omitted for clarity.

Table S2.1 Summary of bond lengths for **1-Δ** and **1-Λ**.

	**1-Δ**	**1-Δ**	**1-Λ**	**1-Λ**
**Bond Length (Å)**				
T (K)	100	293	100	310
Mn–O1	1.8805(18)	1.874(2)	1.8800(15)	1.885(2)
Mn–O2	1.8840(18)	1.883(2)	1.8852(15)	1.877(2)
Mn–N1	1.991(2)	2.052(3)	1.9924(18)	2.058(3)
Mn–N4	1.995(2)	2.036(3)	1.9943(17)	2.047(3)
Mn–N2	2.044(2)	2.106(3)	2.0432(18)	2.137(3)
Mn–N3	2.052(2)	2.123(3)	2.0530(18)	2.119(3)

Table S2.2 Summary of crystallographic details for **1-Δ** and **1-Λ**.

	**1-Λ**	**1-Λ**	**1-Δ**	**1-Δ**
**CCDC Code**	2217782	2217783	2217780	2217781
**Molecular Formula**	$[C_{22} H_{28} N_4 O_2 Mn]^+$ $[C N S]^-$ $C_2 H_6 O$	$[C_{22} H_{28} N_4 O_2 Mn]^+$ $[C N S]^-$ $C_2 H_6 O$	$[C_{22} H_{28} N_4 O_2 Mn]^+$ $[C N S]^-$ $C_2 H_6 O$	$[C_{22} H_{28} N_4 O_2 Mn]^+$ $[C N S]^-$ $C_2 H_6 O$
M_r (g mol-1)	539.57	539.57	539.57	539.57
T (K)	100(2)	310(4)	100(2)	293(2)
**Crystal System**	Monoclinic	Monoclinic	Monoclinic	Monoclinic
**Space Group**	$P2_1$ (#4)	$P2_1$ (#4)	$P2_1$ (#4)	$P2_1$ (#4)
**a** (Å)	7.36734(7)	7.56410(10)	7.36822(8)	7.53420(10)
**b** (Å)	18.9369(2)	18.9752(2)	18.9334(2)	18.9832(3)
**c** (Å)	9.5083(1)	9.70720(10)	9.51000(10)	9.6781(2)
α (°)	90	90	90	90
β (°)	104.513(1)	105.0500(10)	104.500(2)	104.981(2)
γ (°)	90	90	90	90
V (Å^3)	1284.22(2)	1345.49(3)	1284.44(3)	1337.15(4)
Z, Z'	2, 1	2, 1	2, 1	2, 1
**Radiation Type**	Cu-Kα	Cu-Kα	Cu-Kα	Cu-Kα
μ (mm^{-1})	5.235	4.997	5.234	5.028
**Crystal Size** (mm^3)	0.209 × 0.184 × 0.109	0.158 × 0.113 × 0.089	0.249 × 0.198 × 0.136	0.249 × 0.198 × 0.136
**Reflections measured, independent reflections**	26699, 5386	16662, 5597	13414, 5355	14322, 5565
R_{int}	0.0323	0.0195	0.0245	0.0278
**GooF on F^2**	1.034	1.073	1.025	1.032
**Final *R* Indices [I>2σ(I)]**	R_1 = 0.0210, wR_2 = 0.0535	R_1 = 0.0311, wR_2 = 0.0817	R_1 = 0.0263, wR_2 = 0.0690	R_1 = 0.0321, wR_2 = 0.0874
$\Delta\rho_{max}$, $\Delta\rho_{min}$ (e Å^{-3})	0.208, −0.237	0.38, −0.27	0.26, −0.29	0.38, −0.25
**Flack parameter**	-0.006(3)	-0.003(4)	-0.003(2)	-0.003(3)
**Hooft parameter**	-0.0060(12)	0.0050(13)	-0.0037(14)	0.002(2)
**Parson's quotient**	-0.008(2)	-0.004(2)	-0.003(2)	-0.003(3)

S2.1 Intermolecular Interactions

Table S2.3 Summary of hydrogen bonding interactions for **1**.

D–H···A[a]	d(D–H) (Å)	d(H···A) (Å)	d(D···A) (Å)	∠(DHA) (°)
		**1-Δ 100 K**		
N3–H1N3···N5^a	1.00	2.26	3.079(3)	138.0
N2–H1N2···N5^b	1.00	2.05	2.957(3)	149.8
O3–H1O3···S	0.84	2.48	3.317(3)	171.5
		**1-Δ 293 K**		
N3–H1N3···N5^a	1.00	2.29	3.118(5)	139.9
N2–H1N2···N5^b	1.00	2.10	3.000(5)	149.0
O3–H1O3···S	0.84	2.51	3.349(11)	172.8
		**1-Λ 100 K**		
N3–H1N3···N5^a	1.00	2.26	3.083(3)	138.1
N2–H1N2···N5^b	1.00	2.05	2.955(3)	149.6
O3–H1O3···S	0.84	2.49	3.319(2)	172.0
		**1-Λ 310**		
N3–H1N3···N5^a	0.98	2.30	3.121(5)	140.8
N2–H1N2···N5^b	0.98	2.13	3.008(5)	149.1
O3–H1O3···S	0.82	2.57	3.36(1)	161.9

[a]Symmetry operations used to generate equivalent atoms: ^{a} –x,y+1/2,–z+1; ^{b} –x+1,y+1/2,–z+1.

S2.2 Packing

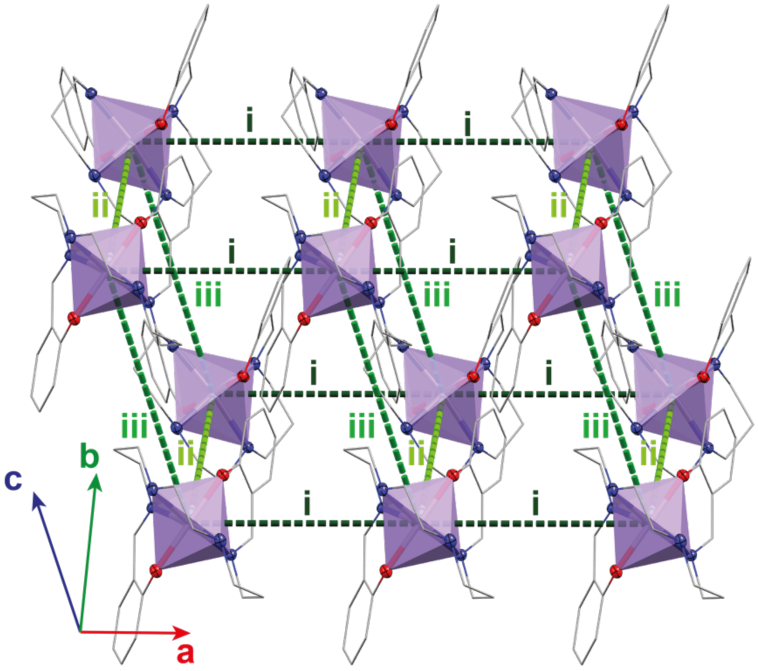

Figure S2.2 View of the packing of the **1-Δ** at 100 K. The Mn^{III} centres are indicated as purple polyhedra. The Mn–Mn distances are labelled according to equivalent distances and are summarised in Table S2.4. Hydrogen atoms, anions and solvent molecules have been omitted for clarity.

Table S2.4 Summary of Mn–Mn distances for **1-Δ** and **1-Λ** at 100 K and room temperature.

Mn–Mn Label[a]	Distance (Å)			
	**1-Δ**		**1-Λ**	
T (K)	100	293	100	310
**i**	7.3682(5)	7.5342(6)	7.3673(4)	7.5641(6)
**ii**	9.4830(5)	9.5034(6)	9.4848(5)	9.4987(5)
**iii**	9.5100(6)	9.6781(6)	9.5083(4)	9.7072(6)

[a]The labels for the Mn–Mn distances correspond with those shown in Figure S2.2.

S2.3 Variable Temperature SCXRD

Variable temperature SCXRD was recorded on a suitable single crystal of **1-Λ** using a Rigaku Oxford Diffraction SuperNova diffractometer with an Atlas CCD detector. The experiments were performed using monochromatic Cu-Kα radiation. The temperature was controlled using an Oxford Instruments Cryojet, with experimental data points recorded every 5 K, with 20 minutes allowed between collection to allow the crystal to fully stabilise in temperature. *CrysAlis*^{PRO} software was used for the data collection and processing.

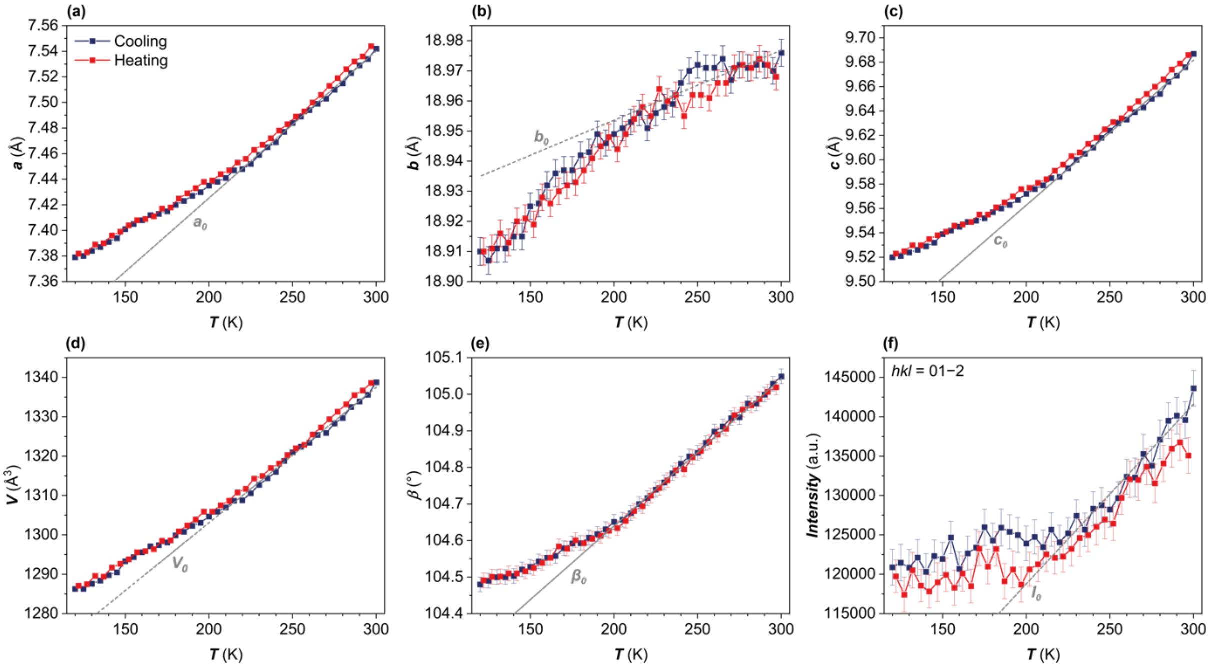

Figure S2.3 Variable temperature determination of unit cell parameters, *a* **(a)**, *b* **(b)**, *c* **(c)**, *V* **(d)**, *β* **(e)**, and the intensity of the *hkl* = 01−2 reflection **(f)**. Extrapolation of the linear fit of the high temperature regions of the parameters (grey line) reveals deviations of reference a_0, b_0, c_0, V_0, β_0 and I_0 parameters at lower temperatures. Standard errors are shown, and where not visible are smaller than the size of the data point.

S2.4 Hirshfeld Surface Analysis

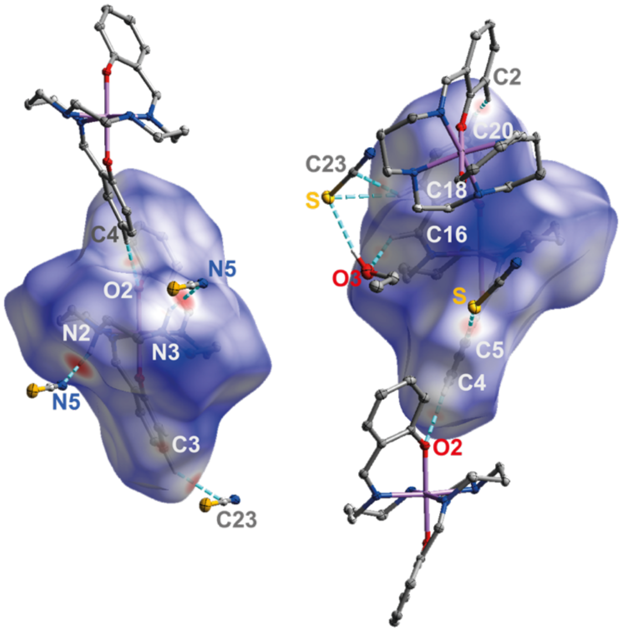

Figure S2.4 Two views of the Hirshfeld surface of **1-Δ** at 100 K mapped with d_{norm} values of −0.4550 to +1.3550 a.u., contacts to atoms outside the surface are shown with hydrogen boning shown in blue. Relevant contact atoms are labelled in colour (outside the surface) and in white (inside the surface).

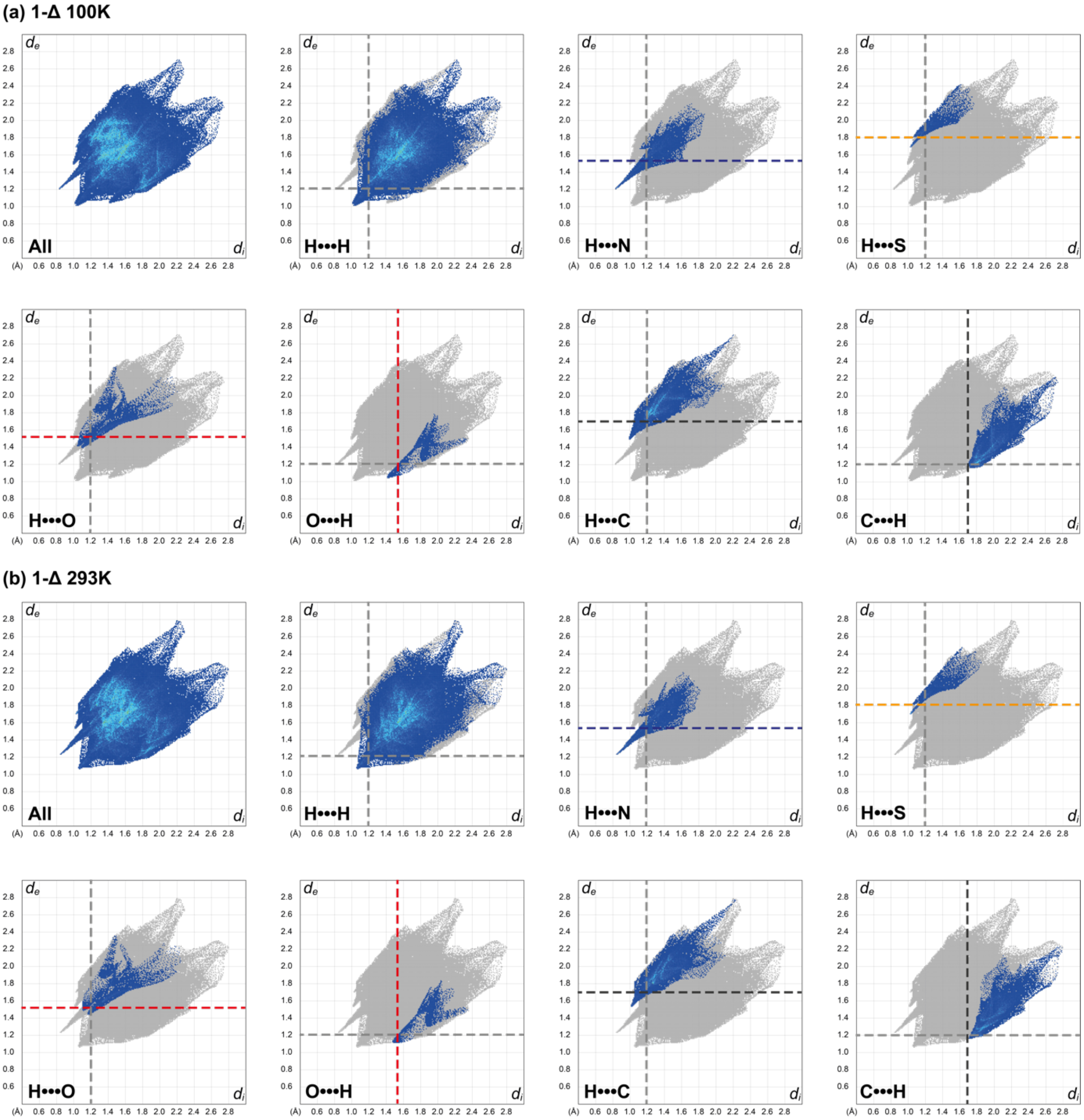

Figure S2.5 *2D* fingerprint plots of **1-Δ** at 100 K for all contacts and delineated into the major atom–atom contributions to the Hirshfeld surface. The dashed lines represent the van der Waals radii of the respective atoms.

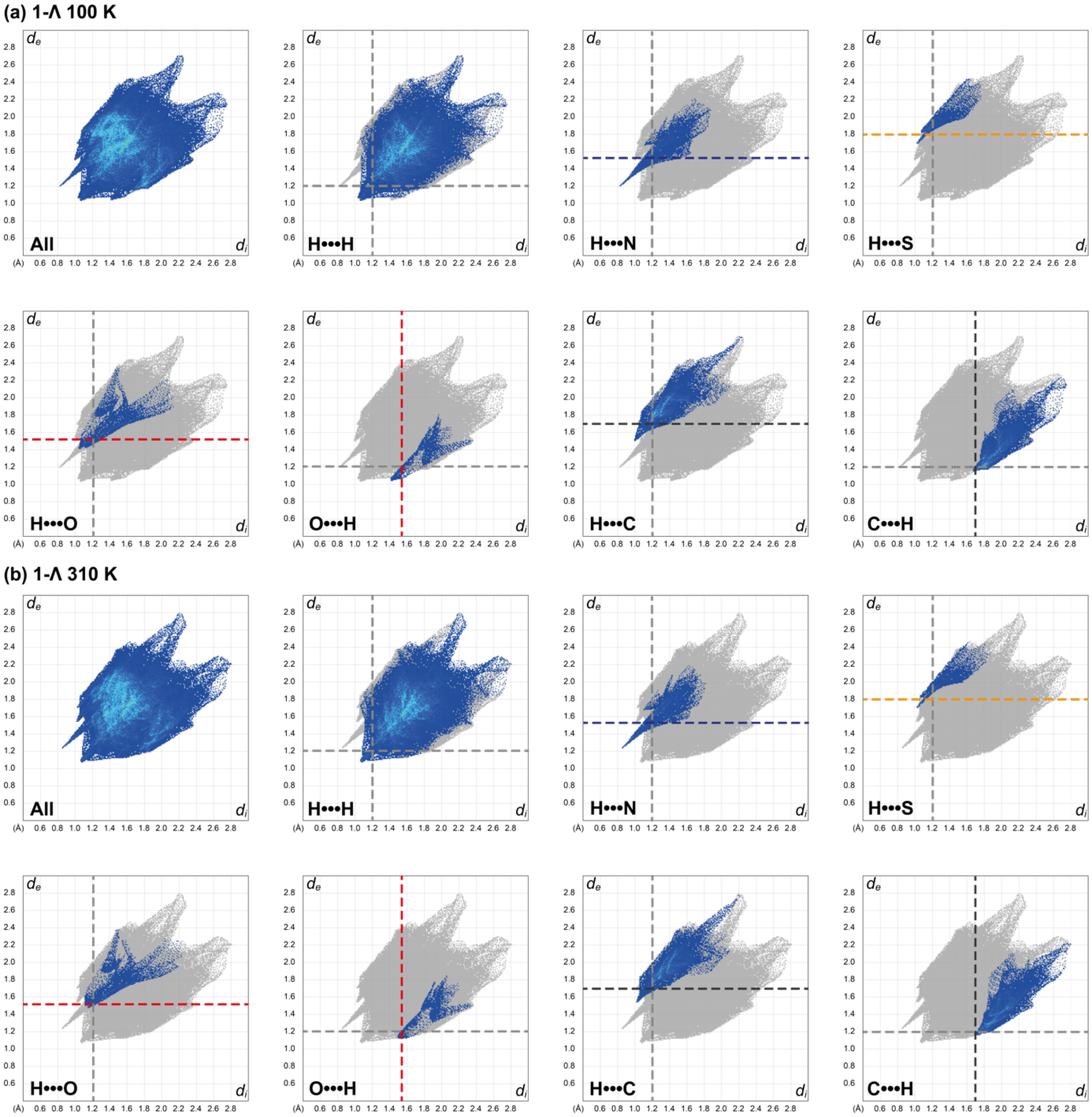

Figure S2.6 *2D* fingerprint plots of **1-Λ** at 100 K for all contacts and delineated into the major atom–atom contributions to the Hirshfeld surface. The dashed lines represent the van der Waals radii of the respective atoms.

S2.5 Database Analysis

A database search of the Cambridge Structural Database (CSD) was carried out using the Conquest program.[1] The search was narrowed down to complexes containing the [Mn^{III}(R-$sal_2$323)]$^+$ cation, where 129 individual complexes were found (some complexes have multiple structures at variable temperature). Of these 18 crystallise in Sohncke space groups (14.0%). Of these only 15 (11.6%) crystallise as potential conglomerates, in most cases only one enantiomer has been identified.

Table S2.5 Summary of entries in the Cambridge Structural Database (CSD) of the [Mn^{III}(R-$sal_2$323)]$^+$ cation which crystallise in Sohncke space groups (where at least one phase is in a Sohncke space group).

CSD Refcode	Molecular Formula	Space Group	Ref.
HAPPAT	[Mn^{III}(4,6-diOMe-$sal_2$323)]Cl	$P2_12_12_1$	[2]
TAQQIP	[Mn^{III}($sal_2$323)]ReO_4	$P2_12_12_1$ / $P2_1$	[3]
BOLJIY/BOLJUK	[Mn^{III}(5-OCF_3-$sal_2$323)][(R,R)-BINOL-B][a]	$I422$	[4]
BOLKAR/BOLJOE	[Mn^{III}(5-OCF_3-$sal_2$323)] [(S,S)-BINOL-B][a]	$I422$	[4]
CAHGUQ[c]	[Mn^{III}(3-OMe-$sal_2$323)][Ni$(dmit)_2$][b]	$P2_1$	[5]
WIQVAT/HAFLIK	[Mn^{III}($sal_2$323)]ClO_4	$P2_12_12_1$	[6]
HOPYAP	[Mn^{III}(napsal$_2$323)]ClO_4	$P2_12_12$	[7]
DAVDEN	[Mn^{III}(4-OMe-$sal_2$323)]ClO_4	$P2_12_12$	[8]
DAVDIR	[Mn^{III}(4-OMe-$sal_2$323)]NO_3	$P2_12_12$	[8]
DAVDOX	[Mn^{III}(4-OMe-$sal_2$323)]Br	$P2_12_12$	[8]
DAVDUD	[Mn^{III}(4-OMe-$sal_2$323)]I	$P2_12_12$	[8]
DAVRAX	[Mn^{III}(4-OMe-$sal_2$323)]BF_4	$P2_12_12$	[8]
MEDCAD	[Mn^{III}(5-Br-$sal_2$323)]I	$P2_12_12$	[9]
MEDDAE	[Mn^{III}(5-I-$sal_2$323)]I	$P2_12_12$	[9]
ZAMDEA[c][d]	[Mn^{III}(3,5-diCl-$sal_2$323)]BPh_4	$P1$	[10]
NEHWAB	[Mn^{III}($sal_2$323)]AsF_6	$P2_12_12_1$	[11]
NUQYIK03[c][d]	[Mn^{III}(3,5-diBr-$sal_2$323)]BPh_4	$P1$	[12]
ZETWOM	[Mn^{III}($sal_2$323)]PF_6	$P2_12_12_1$	[13]

[a]Where [(R,R)-BINOL-B] is (R,R)-bis[1,1′-binaphthyl-2,2′-diolato]boron. [b]With [Ni$(dmit)_2$], where (dmit) is 2-thioxo-1,3-dithiol-4,5-dithiolate. [c]These crystallise as a racemates in a Sohncke space group. [d]Only the low temperature phase is in a Sohncke space group. The complex undergoes a structural phase transition from $Cc \rightarrow Pc \rightarrow P1$.

S3 Magnetic Measurements

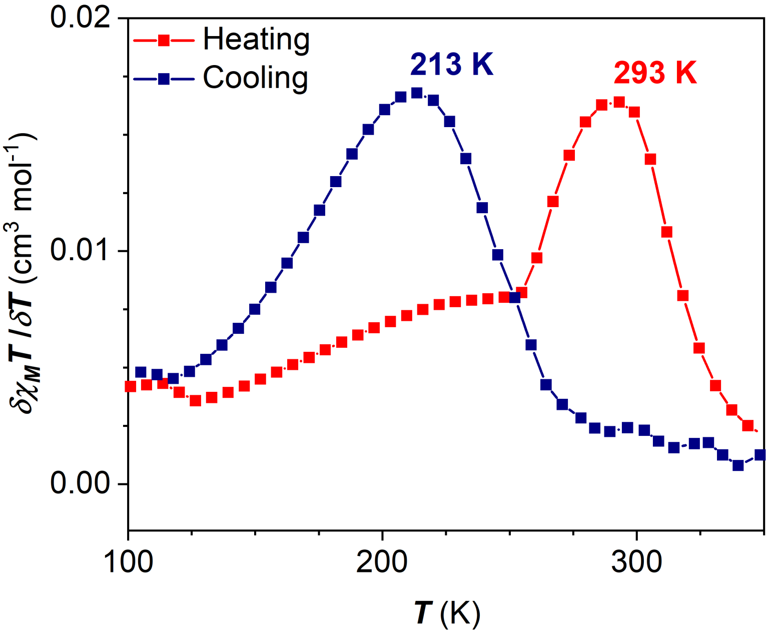

Figure S3.1 Derivative of the magnetic susceptibility with respect to temperature ($\delta\chi_M T/\delta T$) of **1** with the $T_{1/2}$ value for heating and cooling modes labelled.

S4 Quantum Chemistry Calculations

Table S4.1 Optimised geometry of **1-Δ** for the spin triplet (LS, *S* = 1) and spin quintet (HS, *S* = 2) states.

HS, *S* = 2				LS, *S* = 1			
Mn	2.362564	7.640517	4.804078	Mn	2.643653	11.354481	4.403147
O	1.412341	6.625142	3.536609	O	3.648513	12.428894	5.586833
C	0.85806	7.135067	2.44321	C	4.196517	11.999361	6.712947
C	0.79983	6.337444	1.279523	C	4.40865	12.916291	7.767558
H	1.224351	5.333677	1.32422	H	4.100708	13.952092	7.617707
C	0.22787	6.824225	0.109049	C	4.976569	12.501431	8.966268
H	0.21025	6.190794	-0.779655	H	5.114767	13.225636	9.77122
C	-0.325362	8.115348	0.058728	C	5.376444	11.164455	9.151925
H	-0.773365	8.488779	-0.861911	H	5.824734	10.84945	10.09425
C	-0.309791	8.900757	1.202909	C	5.210806	10.258709	8.114942
H	-0.760251	9.895782	1.196535	H	5.544267	9.224344	8.225274
C	0.282215	8.442867	2.40021	C	4.613765	10.643849	6.892614
C	0.169121	9.25698	3.585967	C	4.590713	9.719857	5.793953
H	-0.555765	10.085023	3.542994	H	5.262053	8.853418	5.861669
N	0.812708	9.04939	4.68949	N	3.907496	9.87915	4.694202
C	0.424134	9.793241	5.886874	C	4.296796	9.046266	3.547992
H	-0.292473	10.585585	5.615793	H	3.402652	8.628203	3.070616
H	1.318675	10.2656	6.316568	H	4.917893	8.210966	3.905198
C	-0.210074	8.853185	6.921226	C	5.09151	9.88697	2.541032
H	-0.607202	9.476389	7.736491	H	5.972557	10.316725	3.045057
H	-1.072218	8.341032	6.462859	H	5.467132	9.213641	1.755927
C	0.736053	7.829337	7.542661	C	4.286462	10.985601	1.857131
H	1.625813	8.334016	7.945554	H	4.903909	11.478008	1.087764
H	0.224936	7.328581	8.384454	H	3.404707	10.556643	1.362296
N	1.212671	6.815446	6.576174	N	3.78556	12.016736	2.801517
H	0.398383	6.363372	6.144512	H	4.590448	12.4696	3.252447
C	2.045413	5.777503	7.215456	C	2.985374	13.058461	2.103171
H	1.457892	5.16047	7.915846	H	2.242989	12.534024	1.485052
H	2.825745	6.291526	7.797463	H	3.619545	13.665807	1.439424
C	2.682603	4.902492	6.14739	C	2.297487	13.92297	3.140253
H	3.267787	4.092265	6.613942	H	3.041357	14.441911	3.761879
H	1.901869	4.436167	5.526985	H	1.658711	14.681632	2.66256
N	3.517204	5.732961	5.258175	N	1.502825	13.048731	4.043667
H	4.333221	6.066058	5.784146	H	0.697427	12.683037	3.520493
C	3.985249	4.992768	4.066353	C	1.007207	13.791452	5.229774
H	3.090111	4.702225	3.497333	H	0.399458	14.645445	4.88782
H	4.497242	4.065588	4.380926	H	1.892714	14.187249	5.745074
C	4.925257	5.809705	3.18352	C	0.192456	12.921712	6.179858
H	5.317135	5.138456	2.404615	H	-0.686982	12.508257	5.659692
H	5.791653	6.154986	3.771514	H	-0.184115	13.57037	6.984935

C	4.285464	7.012628	2.475731	C	0.982831	11.776225	6.825067
H	4.996606	7.438181	1.748843	H	1.876123	12.171199	7.323502
H	3.389055	6.685233	1.931122	H	0.358889	11.276574	7.581766
N	3.901199	8.036625	3.447446	N	1.375238	10.799937	5.799268
C	4.550193	9.157013	3.464075	C	0.691292	9.690135	5.752669
H	5.27188	9.360171	2.65751	H	0.018893	9.464038	6.590932
C	4.450529	10.154754	4.500239	C	0.669112	8.780243	4.64218
C	5.061054	11.411574	4.295059	C	0.070972	7.508082	4.794188
H	5.511645	11.614905	3.321108	H	-0.263248	7.208578	5.790087
C	5.09665	12.369111	5.298748	C	-0.093779	6.656123	3.712521
H	5.560058	13.339337	5.120875	H	-0.542553	5.672013	3.847963
C	4.543426	12.063367	6.55434	C	0.307264	7.08504	2.433051
H	4.576997	12.802355	7.356932	H	0.16919	6.427718	1.572603
C	3.952199	10.827535	6.79289	C	0.875773	8.339484	2.247315
H	3.529178	10.584041	7.76839	H	1.185245	8.678121	1.257511
C	3.874875	9.853355	5.773575	C	1.087405	9.206495	3.343396
O	3.30666	8.686813	6.050775	O	1.636615	10.391855	3.129416

Table S4.2 Calculated Gibbs free energy (ΔG_{SCO}) upon SCO from the LS to HS state for the $[Mn^{III}(sal_2323)]^+$ cation.

		ΔE	ΔG_{therm}	ΔG_{SCO}
**$[Mn^{III}(sal_2323)]^+$**	kJ mol^{-1}	3.48579	-15.2986	-11.81281
	kcal mol^{-1}	0.83311	-3.6564	-2.82329

Table S4.3 Energies, oscillator strength, main orbital contributions, and type of transition for **1** calculated with TDDFT using the CAM-B3LYP functional and the def2-TZVPP basis set.

State	$\tilde{\nu}$ (cm^{-1})	λ (nm)	f_{osc}	*Main Transitions*	*Type of transition*
2	17930.6	557.7	0.0029	113a → 116a	*d – d*
3	22696.9	440.6	0.0138	114a → 116a	LMCT
10	31289.1	319.6	0.0292	114a → 117a	LMCT
11	31400.8	318.5	0.0463	111b → 112b	LMCT
16	33771.5	296.1	0.0849	111b → 114b	LMCT
17	34370.9	290.9	0.0141	110b → 113b	LMCT
19	35222.2	283.9	0.0225	110a → 116a	LMCT
20	36510.9	273.9	0.1078	111b → 114b	LMCT
21	36952.1	270.6	0.0124	111b → 115b	LMCT
22	37650.4	265.6	0.0317	110b → 115b	LMCT
25	38812.9	257.6	0.0584	105b → 112b	LMCT
26	39061.4	256	0.0234	105b → 113b*(10%)	LMCT, LLCT
27	39118.2	255.6	0.1790	111a → 116a	LMCT
28	39597.6	252.5	0.0316	109a → 116a	LMCT, LLCT
29	39678.9	252	0.0164	113a → 117a	LMCT, LLCT
31	40498.7	246.9	0.1432	112a → 117a* (12%)	LMCT, LLCT
32	40699	245.7	0.0137	107b → 113b* 10%	LMCT, LLCT
33	41210.7	242.7	0.0919	109b → 112b	LMCT, LLCT

34	41223.2	242.6	0.1794	107b → 112b	LMCT
35	41562.1	240.6	0.0294	105b → 112b* 18%	LMCT
37	42056.5	237.8	0.0122	107b → 113b<br>107b → 115b	LMCT
38	42325.3	236.3	0.0391	107a → 116a	LMCT
40	42857.2	233.3	0.0662	106b → 115b* 12%	LMCT
41	43161.1	231.7	0.0104	110b → 116b	LMCT
43	44024.7	227.1	0.1143	108b → 114b*11%	LMCT, LLCT
46	44732	223.6	0.1008	105b → 114b	LMCT, LLCT
47	44955	222.4	0.1311	110a → 118a*11	LMCT, LLCT
48	44982	222.3	0.0207	106b → 114b	LMCT, LLCT
50	45405.6	220.2	0.1896	109b → 113b	LMCT, LLCT
53	45893.5	217.9	0.1604	110a → 118a	LMCT, LLCT
55	47142.3	212.1	0.0725	111b → 119b*16	LMCT, LLCT
56	47202.5	211.9	0.0427	111b → 119b	LMCT, LLCT
57	47326.8	211.3	0.1838	104b → 115b	LMCT, LLCT
58	47545.3	210.3	0.0316	113a → 123a*11	LLCT
59	47886.3	208.8	0.0251	111b → 119b	LMCT
60	48011.2	208.3	0.0874	111b → 120b	LMCT, LLCT
61	48184.3	207.5	0.0189	111a → 117a	LMCT, LLCT
62	48277.6	207.1	0.0130	109b → 114b*9	LMCT, LLCT
64	48668.1	205.5	0.0933	104b → 114b	LMCT, LLCT

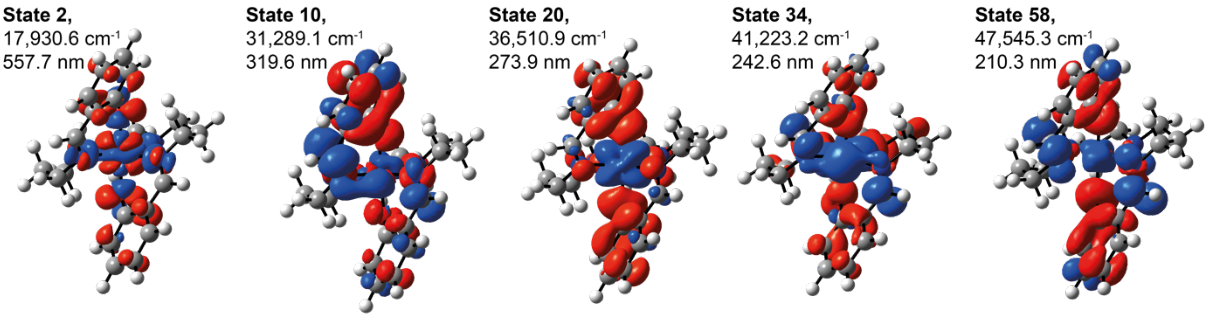

Figure S4.1 TDDFT difference density plots for selected excited states. Isosurface values are plotted at ±0.001 a.u., with positive values in blue and negative values in red.

S5 CRediT Author Statement

Conor T. Kelly: Conceptualization, Methodology, Formal Analysis (lead), Investigation (lead), Data Curation (lead), Writing – Original Draft, Writing – Review & Editing, Visualization. **Ross Jordan:** Investigation, Data Curation. **Solveig Felton:** Resources, Supervision, Funding Acquisition. **Helge Müller-Bunz:** Formal Analysis, Investigation, Data Curation. **Grace G. Morgan:** Conceptualization, Methodology, Resources, Writing – Review & Editing, Supervision (lead), Project Administration, Funding Acquisition (lead).

S6 References

[1] I. J. Bruno, J. C. Cole, P. R. Edgington, M. Kessler, C. F. Macrae, P. McCabe, J. Pearson, R. Taylor, *Acta Crystallogr. Sect. B* **2002**, *58*, 389-397.

[2] K. Pandurangan, A. B. Carter, P. N. Martinho, B. Gildea, T. Lemma, S. Shi, A. Sultan, T. E. Keyes, H. Mueller-Bunz, G. G. Morgan, *Magnetochemistry* **2022**, *8*, 8.

[3] E. Dobbelaar, V. B. Jakobsen, E. Trzop, M. Lee, S. Chikara, X. Ding, H. Müller‐Bunz, K. Esien, S. Felton, M. A. Carpenter, E. Collet, G. G. Morgan, V. S. Zapf, *Angew. Chem. Int. Ed.* **2022**, *61*, e202114021.

[4] V. B. Jakobsen, L. O'Brien, G. Novitchi, H. Müller‐Bunz, A. L. Barra, G. G. Morgan, *Eur. J. Inorg. Chem.* **2019**, *2019*, 4405-4411.

[5] S. Wang, Y. H. Li, W. Huang, *Eur. J. Inorg. Chem.* **2015**, *2015*, 2237-2244.

[6] A. Panja, N. Shaikh, S. Gupta, Ray J. Butcher, P. Banerjee, *Eur. J. Inorg. Chem.* **2003**, *2003*, 1540-1547.

[7] A. Barker, C. T. Kelly, I. A. Kuhne, S. Hill, J. Krzystek, P. Wix, K. Esien, S. Felton, H. Muller-Bunz, G. G. Morgan, *Dalton Trans.* **2019**, *48*, 15560-15566.

[8] I. A. Kühne, A. Ozarowski, A. Sultan, K. Esien, A. B. Carter, P. Wix, A. Casey, M. Heerah-Booluck, T. D. Keene, H. Müller-Bunz, S. Felton, S. Hill, G. G. Morgan, *Inorg. Chem.* **2022**, *61*, 3458-3471.

[9] C.-Y. Qin, S.-Z. Zhao, H.-w. Zhou, Y.-H. Li, S. Wang, *Polyhedron* **2022**, *222*, 115896.

[10] S. Ghosh, S. Bagchi, M. Das, S. Kamilya, A. Mondal, *Dalton Trans.* **2020**, *49*, 14776-14780.

[11] S. Wang, Y.-J. Li, F.-F. Ju, W.-T. Xu, K. Kagesawa, Y.-H. Li, M. Yamashita, W. Huang, *Dalton Trans.* **2017**, *46*, 1163-1177.

[12] V. B. Jakobsen, E. Trzop, L. C. Gavin, E. Dobbelaar, S. Chikara, X. Ding, K. Esien, H. Müller‐Bunz, S. Felton, V. S. Zapf, E. Collet, M. A. Carpenter, G. G. Morgan, *Angew. Chem. Int. Ed.* **2020**, *59*, 13305-13312.

[13] P. N. Martinho, B. Gildea, M. M. Harris, T. Lemma, A. D. Naik, H. Müller‐Bunz, T. E. Keyes, Y. Garcia, G. G. Morgan, *Angew. Chem. Int. Ed.* **2012**, *51*, 12597-12601.
